# Differences in anterior chamber depth in keratoconus patients with binocular very asymmetry ectasia

**DOI:** 10.1186/s12886-024-03353-5

**Published:** 2024-02-26

**Authors:** Zizhen Wang, Haowen Ma, Yu Zhang, Yifei Yuan, Yan Liu, Yueguo Chen

**Affiliations:** 1 Department of Ophthalmology, Beijing Key Laboratory of Restoration of Damaged Ocular Nerve, Peking University Third Hospital, 49 North Garden Road, Haidian District, 100191 Beijing, People’s Republic of China; 2 Peking University Health Science Center, 38 North Garden Road, Haidian District, 100191 Beijing, People’s Republic of China; 3 Peking University Institute of Laser Medicine, Beijing, China

**Keywords:** Keratoconus, Anterior chamber depth, Corneal magnification effect, Very asymmetry ectasia

## Abstract

**Background:**

To evaluate the difference in anterior chamber depth (ACD) between two eyes among keratoconus patients with binocular very asymmetric ectasia (VAE) and to explore the influencing factors.

**Methods:**

The corneal curvature and ACD in both eyes of patients with VAE were measured by Sirius (version 3.2, CSO, Italy) at the following points: corneal vertex, maximum curvature (apex), and the 1.5 mm, 2.5 mm, and 3.5 mm superior-, inferior-, nasal-, temporal-paracentral from center. The mean pupil power (MPP) and corneal morphology parameters were also measured. Correlations between ACD and curvature and morphology parameters were analyzed by linear regression.

**Results:**

172 eyes of 86 patients (9 to 45 years) were classified into the VAE-N (*n* = 86) group and the VAE-E group (*n* = 86) based on the corneal morphology. The central (3.32 ± 0.27 mm versus 3.43 ± 0.29 mm, *P* < 0.001) and paracentral ACDs increased significantly in the VAE-E group, and the corneal morphology parameters were also significantly higher. The central ACD was significantly correlated with the MPP (*r* = 0.465), KVf/b (Keratoconus Vertex front/back) (*r* = 0.306, *r* = 0.327), and BCVf/b (Baiocchi Calossi Versaci front/back) (*r* = 0.356, *r* = 0.416). Linear regression showed good relationships between △ACD and △MPP (R^2^ = 0.429) and △KVf/b (R^2^ = 0.504, R^2^ = 0.536).

**Conclusions:**

The ACD was larger in the VAE-E group. The difference in ACD between the VAE-E and VAE-N groups was significantly correlated with corneal curvature and the extent of corneal elevation, indicating the influences of both the corneal magnification effect and corneal ectasia on ACD.

## Introduction

Keratoconus is a noninflammatory ectatic corneal disorder that is commonly bilateral and usually asymmetrical, in which the ectatic cornea becomes conical in shape [1]. It is usually associated with irregular astigmatism and visual impairment [2]. It typically presents in adolescence and progresses until the third or fourth decade of life and is one of the most common reasons for keratoplasty in the developed world; however, this demand is decreasing with the onset of corneal collagen crosslinking [3, 4].

Most relevant research focuses on the diagnosis and treatment of keratoconus, and there are few studies of anterior chamber characteristics in the eyes of patients with keratoconus [5]. The structure of the anterior chamber is mainly determined by the posterior surface of the cornea and the anterior surface of the crystalline lens. Some studies have reported that keratoconus and keratoplasty might contribute to alterations in anterior chamber parameters [6, 7].

Because keratoconic patients tend to have high myopia and myopic astigmatism, the motivation of such patients among all candidates for corneal refractive surgery has been shown to be relatively high. However, due to their poor corneal thickness, the implantation of intraocular collamer lenses (ICLs) is more suitable and safer than laser-assisted surgeries [8, 9]. The accuracy of the IOL power calculation is of great significance for the postoperative visual quality of cataract patients. Currently, widely used calculation formulas, such as the Kane formula, BUII, Olsen (four-factor), Haigis, Holladay 2 and Barrett True-K, involve measurements of axial length, keratometry, anterior chamber depth (ACD), lens thickness and central corneal thickness (CCT) [10–12]. Thus, anterior chamber parameters are important factors for the evaluation of intraocular lens (IOL) implantation surgery [13–15], and it is clinically important to effectively evaluate changes in the anterior chamber after the development of keratoconus.

We noticed that the corneal magnification effect may cause changes of anterior chamber parameters measured optically through the cornea [16, 17]. Thus, in this study, we aimed to investigate the changes in anterior chamber depth (ACD) in keratoconus patients with binocular VAE (one eye appears normal and the other is keratoconus) and to explore whether corneal magnification effect caused by cornea ectasia leads to errors in the anterior chamber depth measurement.

## Methods

This was a self-controlled retrospective study that involved 172 eyes of 86 patients (age range 9 to 45 years, mean 23.63 ± 6.76 years). The main reasons for visiting our clinic were progressive aggravation of myopia or astigmatism, poorly corrected visual acuity found during optometry or ectatic corneal topography and tomography in screening examination before refractive surgery. The study was conducted in accordance with the tenets of the Declaration of Helsinki (Fortaleza, 2013) and was approved by the local ethics committee (Peking University Third Hospital Medical Science Research Ethics Committee). Written informed consent was obtained from every patient, and for human participants that are minors, written informed consent was obtained from their legal guardians.

The exclusion criteria for the study included a history of contact lens wear, active eye infection or inflammation, ocular operation or trauma history and severe systemic diseases.

### Case grouping

Based on corneal topography and tomography examination results, all eyes were divided into a very asymmetric ectasia-normal (VAE-N) group (*n* = 86) and a very asymmetric ectasia-ectasia (VAE-E) group (*n* = 86) by an experienced ophthalmologist (YG Chen).

### Assessment

A complete ophthalmological examination was performed in all patients, including measurements of uncorrected visual acuity (UCVA), best corrected visual acuity (BCVA), intraocular pressure (IOP), slit-lamp examination, and fundoscopy.

The following parameters were assessed using Sirius combined topography and tomography (version 3.2, CSO, Italy) in the VAE-N and VAE-E groups: central corneal thickness (CCT), mean pupil power (MPP), maximal anterior corneal surface curvature (Kmax), flat meridian corneal curvature (K1), steep meridian corneal curvature (K2), spherical equivalent (SE), corneal astigmatism, thinnest corneal thickness (TCT), anterior chamber angle (ACA), anterior chamber volume (ACV), ACD at central, superior-, inferior-, nasal- and temporal-paracentrally, and at the point of Kmax, corneal volume within a diameter equal to 10 mm (CV). Ocular axial lengths were obtained by ZEISS IOL MASTER 700.

The symmetry index of front and back corneal curvature (SIf & SIb) is the difference between the mean value of tangent curvature in two 3.0-mm diameter circular regions above and below the center of anterior and posterior surfaces of the cornea. It is a vertical asymmetry index, with a positive value indicating steeper in the lower hemisphere and a negative value indicating steeper in the upper hemisphere. BCV (ф=8.00 mm) is an abbreviation of author Baiocchi Calossi Versaci, who used the aberration (including Coma, Trefoil, and spherical aberration) of Zernike polynomial decomposition in the central 8-mm diameter analysis area. BCVf (Baiocchi Calossi Versaci front) = F ($$ {C}_{3f}^{\pm 1}$$, $$ {C}_{3f}^{\pm 3}$$, $$ {C}_{4f}^{0}$$). BCVb (Baiocchi Calossi Versaci back) = F ($$ {C}_{3b}^{\pm 1}$$, $$ {C}_{3b}^{\pm 3}$$, $$ {C}_{4b}^{0}$$). $$ {C}_{3}^{\pm 1}$$ represents the horizontal and vertical coma aberration, $$ {C}_{3}^{\pm 3}$$ represents the horizontal and vertical trefoil aberration, and $$ {C}_{4}^{0}$$ represents the the primary spherical aberration. The vector sum of BCV in anterior and posterior surfaces of the cornea presented is in micrometers (µm) and its direction. BCV is used to evaluate the state of corneal protrusion, which means the deviation of the aberration center toward the direction of corneal protrusion. This is the result of an unbalanced increment of corneal aberration in the horizontal and vertical directions. BCV of anterior and posterior surfaces in keratoconus is correlated, and overlap of axes of these two vectors can create a larger BCV. In contrast, axes are not correlated in non-keratoconus eyes with abnormal morphology, resulting in a decrease in the overall BCV value. Keratoconus Vertex front & back (KVf & KVb) reflects the difference between the mean values of the results measured within two apex-centered 8-mm diameter areas on anterior and posterior surfaces of the cornea and on the asphero-toric best fit reference surface. KVf and KVb reflect the height difference between the measured cornea and reference database, indicating the extent of corneal protrusion and height of the corneal apex. Compared with the traditional height map, which only considers the cornea itself, the best-fit spherical reference surface varies with different corneas; thus, KVf and KVb are more applicable, and the values are more comparable.

### Statistical analysis

Data are presented as the mean ± standard deviation.

All statistical analyses were performed using IBM SPSS Version 24 statistic software. The Shapiro-Wilk W test was used to assess the normality of the data. A paired *t-test* was used to compare variables, including the baseline characteristics of the patients and the corneal topography parameters. Pearson’s correlation coefficient was used to determine the degree of correlation between corneal morphology parameters and ACD. Correlations between corneal topography parameters, △MPP and △ACD were tested using linear regression analysis. Statistical significance was set at *P* < 0.05.

## Results

**Fig. 1:**
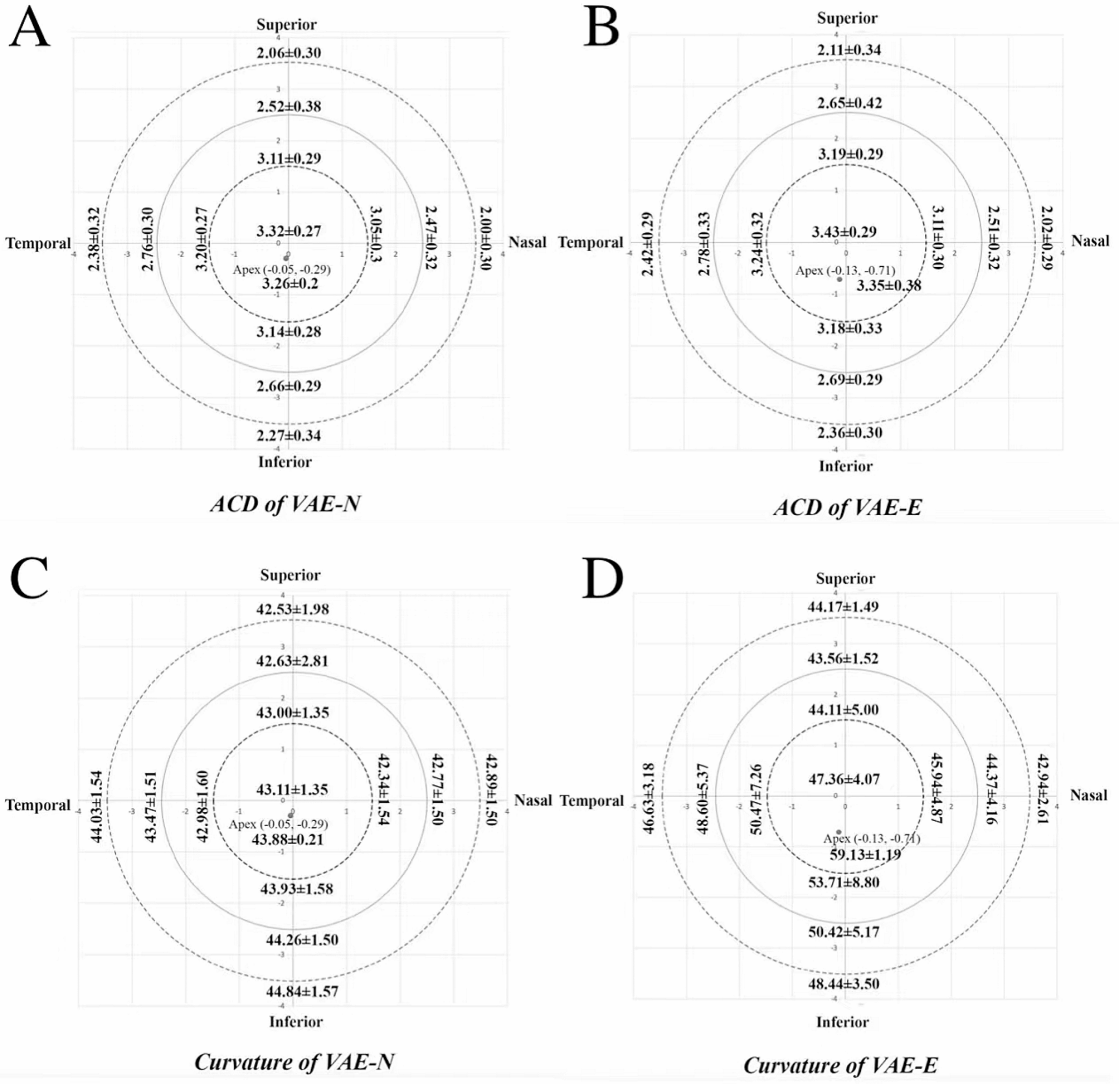
(**A**, **B**, **C**, **D**): ACD and corneal curvature at all measurement points (center, apex, 1.5 mm, 2.5 mm, 3.5 mm superior-paracentral, inferior-paracentral, nasal-paracentral, temporal-paracentral) in the VAE-N and the VAE-E groups

Figure 1; Table 1 show the ocular axial length, ACV, ACD and corneal curvature at all measurement points (1.5 mm, 2.5 mm, 3.5 mm superior-paracentral, inferior-paracentral, nasal-paracentral, temporal-paracentral) and other corneal parameters in the VAE-E (Ectasia) and the VAE-N (Normal) groups. Eyes in VAE-E group were found to present significant higher values of Kmax, MPP and corneal astigmatism than those in the VAE-N group, while the CCT and TCT in the VAE-E group were significantly thinner than those in the VAE-N group. There was no significant difference in ocular axial length and corneal volume between the two groups.

**Table 1:** Anterior chamber and corneal morphology by group

Items	VAE-N(*n* = 86)	VAE-E(*n* = 86)	*P* value
ACA (°)	45.14 ± 5.43	45.12 ± 5.33	0.968
Central ACD (mm)	3.32 ± 0.27	3.43 ± 0.29	< 0.001
ACV (mm^3^)	188.49 ± 28.08	191.05 ± 28.36	0.006
Ocular axial length (mm)	25.59 ± 0.17	25.59 ± 0.17	0.257
Spherical equivalent (D)	-5.10 ± 3.04	-4.52 ± 2.95	< 0.001
Astigmatism (D)	-3.80 ± 2.19	-6.73 ± 4.27	< 0.001
Kmax (D)	45.45 ± 1.82	59.13 ± 10.19	< 0.001
CCT (µm)	526.49 ± 30.74	484.92 ± 41.79	< 0.001
TCT (µm)	520.85 ± 30.42	469.27 ± 43.01	< 0.001
Corneal volume (mm^3^)	56.90 ± 3.13	56.82 ± 3.15	0.669

Abbreviations: ACA = anterior chamber angle, ACV = anterior chamber volume, ACD = anterior chamber depth, CCT = central corneal thickness, TCT = thinnest corneal thickness

The values are presented as median (non-normal distribution) or mean ± standard deviation (normal distribution)

We measured the ACA, ACV, ACD and corneal curvature at the corneal center and 1.5 mm, 2.5 and 3.5 mm paracentrally (four directions at each radius: superior, inferior, nasal and temporal) in both groups. The ACD at the corneal apex in the eyes of patients in the VAE-E group and the corresponding ACD in the VAE-N group were measured as well. As shown in Fig. 1, the ACV, ACD and the corresponding corneal curvature at the corneal center and all four directions at different radii in the eyes of patients in the VAE-E group were all significantly higher than those in the VAE-N group. No significant differences in ACA between the two groups were found (*P* = 0.968).

We measured the following morphological parameters in the VAE-N and VAE-E groups using Sirius: Symmetry Index front/back (SIf, SIb), Baiocchi Calossi Versaci index front/back (BCVf, BCVb), and Keratoconus Vertex front/back (KVf, KVb), as shown in Table 2.

**Table 2:** Comparison of corneal morphology between VAE-N and VAE-E eyes

Index	VAE-N(*n* = 86)	VAE-E(*n* = 86)
SIf (D)	0.41 ± 0.68	5.90 ± 5.03^***^
SIb (D)	0.24 ± 0.20	2.48 ± 6.80^**^
BCVf (µm)	0.30 ± 0.25	3.43 ± 2.65^***^
BCVb (µm)	0.43 ± 0.35	3.48 ± 2.12^***^
KVf (µm)	4.80 ± 2.28	38.27 ± 27.13^***^
KVb (µm)	14.56 ± 8.68	86.81 ± 55.25^***^

Abbreviations: SIf = Symmetry Index front, SIb = Symmetry Index back, BCVf = Baiocchi Calossi Versaci index front, BCVb = Baiocchi Calossi Versaci index back, KVf = Keratoconus Vertex front, KVb = Keratoconus Vertex back

^***^P < 0.001 and ^**^P < 0.01 (VAE-N versus VAE-E, paired t-test)

According to the statistical results, SIf & SIb, BCVf & BCVb, KVf & KVb in the VAE-E group were all significantly higher in the VAE-N group (*P* < 0.005), which indicated that the curvature of the anterior and posterior surfaces of the cornea in the eyes of patients in the VAE-E group was significantly asymmetric and that both surfaces in the VAE-E group underwent a certain degree of protrusion and elevation.

To explore the changes in anterior chamber depth in eyes with keratoconus and possible influencing factors, we investigated the effects of curvature and corneal morphology on anterior chamber depth (Tables 3 and 4).

**Table 3:** Correlations between △ACD and △Curvature at different measure points

Index		r	*P*
Superior	1.5 mm paracentral	0.291^**^	0.007
	2.5 mm paracentral	0.216^*^	0.045
	3.5 mm paracentral	0.202	0.069
Inferior	1.5 mm paracentral	0.086	0.433
	2.5 mm paracentral	0.143	0.188
	3.5 mm paracentral	0.573^***^	< 0.001
Nasal	1.5 mm paracentral	0.131	0.231
	2.5 mm paracentral	0.192	0.076
	3.5 mm paracentral	0.172	0.112
Temporal	1.5 mm paracentral	0.277^**^	0.010
	2.5 mm paracentral	0.132	0.226
	3.5 mm paracentral	0.322^**^	0.003
At VAE-E apex		0.219^*^	0.042
Central		0.655^***^	< 0.001

^***^P < 0.001, ^**^P < 0.01 and ^*^P < 0.05

**Table 4:** Correlations between central ACD and corneal morphology index

Index	VAE-N(*n* = 86)	VAE-E(*n* = 86)
	r	r
SIf (D)	0.176	0.155
SIb (D)	0.160	0.084
BCVf (µm)	0.052	0.306^***^
BCVb (µm)	0.027	0.327^**^
KVf (µm)	0.043	0.356^***^
KVb (µm)	0.213^*^	0.416^***^
MPP(D)	0.061	0.465^***^

Abbreviations: SIf = Symmetry Index front, SIb = Symmetry Index back, BCVf = Baiocchi Calossi Versaci index front, BCVb = Baiocchi Calossi Versaci index back, KVf = Keratoconus Vertex front, KVb = Keratoconus Vertex back, MPP = mean pupil power

^***^P < 0.001, ^**^P < 0.01 and ^*^P < 0.05 (VAE-N versus VAE-E, paired t-test

In terms of refractive power, statistically significant positive correlations were found between changes in anterior chamber depth (△ACD) and changes in corneal curvature (△Curvature) at the corneal center, 1.5 and 2.5 mm superior-paracentral, 3.5 mm inferior-paracentral, 1.5 and 3.5 mm temporal-paracentral, and at the cornea apex in the VAE-E group. No significant correlations were found between △ACD and △Curvature in other directions, but the positive r values indicated that they were well consistent. The central ACD was significantly higher in the VAE-E group than in the VAE-N group and had a strong correlation with the MPP (*r* = 0.465, *P* < 0.001) and corneal morphology parameters (BCVf, BCVb, KVf, KVb). In contrast, no significant correlation was found between central ACD and MPP (*r* = 0.061, *P* = 0.576) or any other corneal morphology parameters in the VAE-N group.

**Fig. 2:**
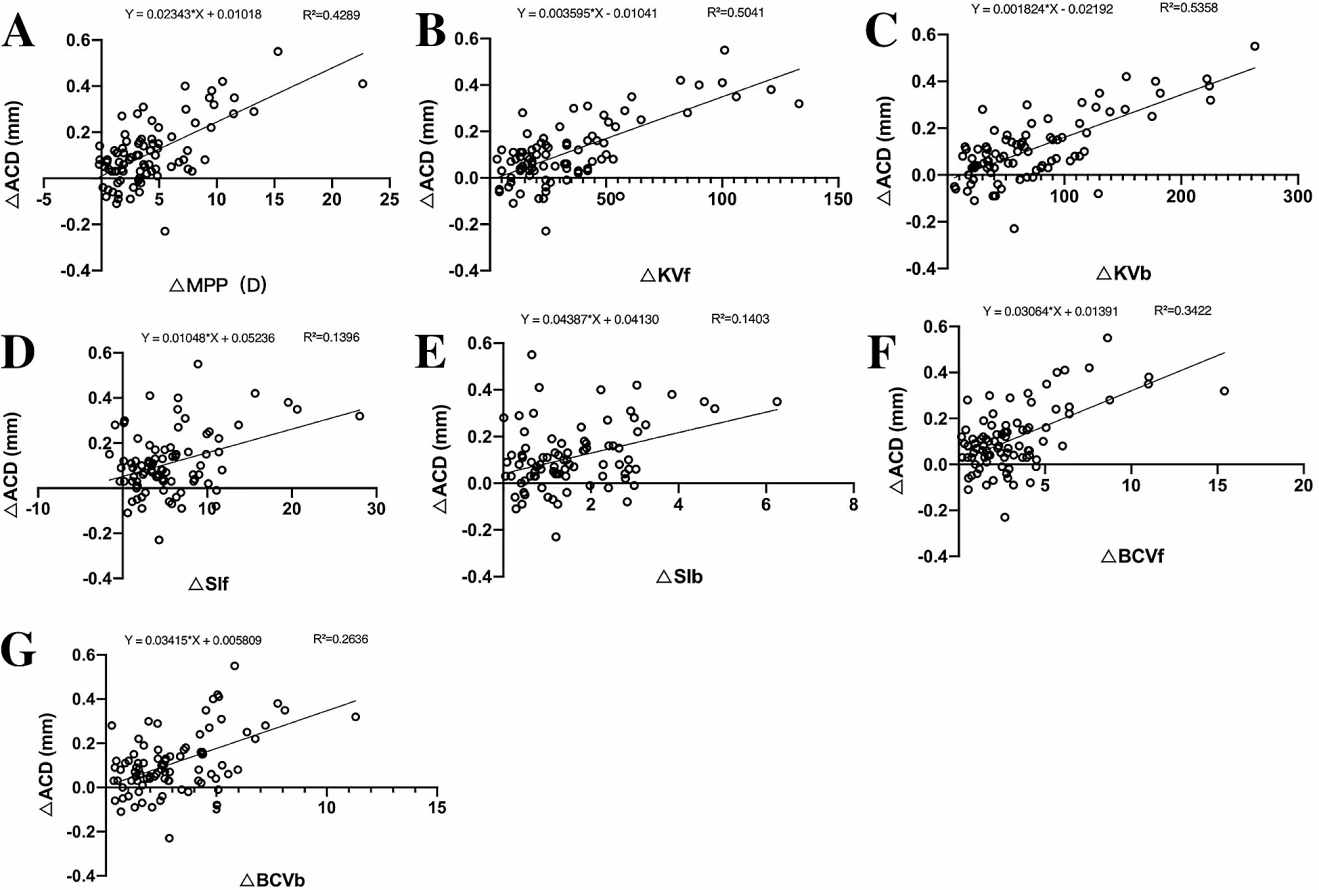
(**A**, **B**, **C**, **D**, **E**, **F**, **G**): Linear correlation between △MPP, △KVf, △KVb, △SIf, △SIfb, △BCVf, △BCVb and △ACD

In terms of corneal morphology, the central ACD in the VAE-E group was significantly correlated with BCV, SI, KV and MPP (Table 4). Linear fit was performed between △ACD and △MPP, △SIf, △SIb, △BCVf, △BCVb, △KVf, and △KVb. Figure 2 shows that △KVf (R^2^ = 0.504, *P* < 0.0001), △KVb (R^2^ = 0.536, *P* < 0.0001) and △MPP (R^2^ = 0.429, *P* < 0.0001) had a greater influence on △ACD..

## Discussion

Anterior chamber morphology plays an important role in intraocular lens implantation, glaucoma screening and cataract IOL power calculation formulas [8–12]. However, clinical studies on the morphological changes of the anterior chamber with the progression of keratoconus are limited, and no clear conclusions have been drawn. To the best of the author’s knowledge, this is the first self-controlled study to investigate the changes in anterior chamber morphology between the affected eye (VAE-E) and the normal eye (VAE-N) in the same patient.

Anterior chamber morphologic parameters, including ACV, ACD and ACA, can be measured by a three-dimensional anterior segment analysis system[ [18]]. In this study, the Sirius system was used to measure parameters of the anterior chamber. A wide variety of instruments have been used to evaluate the anterior segment, including Placido disc corneal topography, Scheimpflug imaging, scanning slit topography, and optical coherence tomography (OCT) [19]. The Sirius system combines the advantages of Placido disc curvature measurement of the anterior surface of the cornea and rotating Scheimpflug camera elevation measurement by imaging the anterior and posterior surface of the cornea, making it possible to provide anterior segment tomography and corneal topographic evaluation in seconds and to improve the accuracy of measurement [20]. In addition, the consistency of Sirius with that of a previous Scheimpflug camera has been confirmed in a number of previous studies [21].

We found that the CCT and TCT were significantly thinner in the eyes of patients in the VAE-E group than in the VAE-N group, while the MPP and Kmax were significantly higher. A significant decrease in the CCT and TCT in one of the two eyes of the same patient, accompanied by an increase in the maximum corneal curvature and MPP, may be indicative of the development of keratoconus, which can be combined with corneal topography results to assist the diagnosis of keratoconus. The CCT and TCT are also important reference parameters in preoperative examinations of refractive surgery to detect keratoconus or any other types of keratectasia [22].

The central ACD was found to be significantly deeper (3.43 ± 0.29 mm versus 3.32 ± 0.27 mm, *P* < 0.001) and the ACV significantly larger (188.49 ± 28.08 mm^3^ versus 191.05 ± 28.36 mm^3^, *P* = 0.006) in the VAE-E group than in the VAE-N group, while no statistically significant difference was found in the ACA, which is consistent with the findings of previous studies [4, 23–27]. The anterior chamber was further found to be either significantly or slightly deeper in the VAE-E group than in the VAE-N group at all measurement points (1.0 mm, 2.0 mm, 3.0 mm superior-, inferior-, nasal-, temporal-paracentral). Since this study was based on a self-controlled design, the structures and ACDs of two eyes of the same patient were expected to be symmetric, so was the anterior chamber depth. Therefore, it is worthwhile for us to explore the possible reasons for changes in ACD. Two hypotheses are proposed here: ①the magnification effect of the cornea leads to systematic error of the measurement of corneal tomography; and ② the anterior chamber does have structural changes.

In both groups, the central ACD was the deepest and became shallower with the increase in the paracentral radius in the periphery. Nevertheless, paracentral ACD in the VAE-E group was always deeper than that in the VAE-N group. Kovacs et al. also evaluated the ACD 1 mm, 2 and 3 mm inferior-paracentral in the keratoconus group and in the control group and found that the ACD was significantly deeper in the former, which was consistent with our results [24]. Abolbashari et al. once reported a correlation between corneal curvature and anterior segment parameters in keratoconus patients, such that the aggravation of keratoconus and the increase in corneal curvature led to an increase in the ACD, and the change in the ACD at the corneal center was greater than that at the periphery [28]. In our study, we noticed that the MPP was less than the Kmax (47.36 ± 4.07 D vs. 59.13 ± 1.19 D), however, the ACD at the corneal center was deeper than that at the maximum curvature point (3.43 ± 0.29 mm vs. 3.35 ± 0.38 mm). Considering that the MPP was less than the Kmax while the ACD in the central cornea was deeper than at the maximum curvature point, we supposed that keratoconus (i.e., point of the maximum curvature) always happens at the temporal paracentrally [1], which was consistent with the perspective of Abolbashari et al. mentioned above. Ort A et al. measured the anterior segment using Sirius and found that 6 months after penetrating keratoplasty (PK), Kmax decreased by 19.6 D (30%), and the average Km decreased by 19.1 D (31.8%), accompanied by a significantly shallower ACD, which decreased by 0.91 mm (23%) from 3.92 ± 0.47 mm preoperatively to 3.01 ± 0.55 mm postoperatively [6]. The finding that the ACD became shallower when corneal curvature decreased after PK is suggestive of the corneal magnification effect [29]. According to Nawa et al., who measured the anterior segment using Obscan, the ACD in patients with myopia decreased after FS-LASIK as a result of the decreased magnification effect of the cornea, which made the overall measurements of the intraocular structure smaller [16, 17]. Similarly, the increased corneal curvature and magnification effect with the progression of keratoconus may cause the overall measurement of intraocular structure to be larger and the significant deepening of ACD in patients with monocular keratoconus. According to Nawa’s conclusion that the magnification rate of the cornea would be reduced by 0.35% for every 1.0-D correction, the central ACD in eyes with keratoconus was supposed to be 3.37 mm. However, the actual measured value of the central ACD in the VAE-E group was 3.43 ± 0.29 mm, which was deeper than the theoretical value. Different magnification effects of the measurement devices (Obscan vs. Sirius) are considered to be a possible reason, and the manufacturer of Sirius may have also implemented a certain correction to corneal magnification.

Corneal morphological parameters, including SIf & SIb, BCVf &BCVb, and KVf & KVb, were all significantly higher in the VAE-E group than in the VAE-N group, indicating that the inferior cornea was much steeper than the superior part, and there were certain degrees of ectasia, bulging and elevation on both anterior and posterior corneal surfaces in the keratoconus eyes. Furthermore, no significant correlations were found between SIf (*P* = 0.105) or SIb (*P* = 0.141) and ACD, while BCV and KV were significantly correlated with ACD in the VAE-E group, indicating that corneal ectasia as well as the elevation of anterior and posterior surfaces of the cornea might cause the deepening of ACD. This is consistent with the findings of Zuzana Schlegel et al., who suggested that the anterior and posterior corneal surfaces in eyes with suspected keratoconus were significantly elevated compared with normal eyes [30]. Linear fits were further examined between the corneal morphological parameters and △ACD. △KVf (R^2^ = 0.504) and △KVb (R^2^ = 0.536) were found to have greater effects on △ACD than SIf/b and BCVf/b, suggesting that the deepening of ACD was more related to corneal elevation. This is also consistent with the findings of Illes Kovacs et al., who measured ACD in 41 normal eyes and 70 keratoconus eyes using Pentacam and found that deepening of the ACD was significantly correlated with the extent of posterior elevation [5]. Enric Mas-Aixala et al., who compared 84 conical eyes and 49 normal eyes, suggested that deepening of the ACD in keratoconus was mainly due to the morphological changes in the corneal and limbus sclera, which increased the distance from the endpoint of the sagittal height at the limbus-to-limbus chord to the anterior surface of the lens and similar degrees of deepening in all meridians, which was consistent with our results [23]. Emre et al. studied 216 conical eyes and found a significant correlation between ACD and the severity of keratoconus. They believed that the increase in ACD was due to corneal protrusion [7], which echoed our findings that BCVf/b and KVf/b in the VAE-E group were significantly higher than those in the VAE-N group and that positive correlations were found between △BCV, △KV and △ACD. In summary, corneal elevation and ectasia caused by keratoconus do play a role in deepening the ACD..

## Conclusion

In conclusion, patients selected for this study who had monocular detectable keratoconus are a valuable group of participants. A self-controlled study was conducted to explore the morphological changes in the anterior chamber after the development of keratoconus, in which confounding factors such as age and gender could be excluded. According to our results, the ACD was significantly deeper in eyes with keratoconus, with the deepest part located in the central cornea and varying degrees of deepening in the periphery. The change in ACD was significantly correlated with corneal curvature and the extent of anterior and posterior corneal elevation. We propose that the increased magnification effect caused by increased corneal curvature would result in a larger measurement value of ACD, and the elevation of anterior and posterior surfaces of the cornea was also involved. It’s worthwhile to further study the changes in the ACD in keratoconus. This study aimed to increase awareness among ophthalmic surgeons that the real ACD might be shallower than the measurement value in keratoconus patients, which is of critical importance for the surgical treatment and diagnosis of other ophthalmic diseases. In addition, dynamic changes in anterior chamber depth can also be used for monitoring the development of the disease in keratoconus patients.

## Data Availability

The datasets used and/or analysed during the current study are available from the corresponding author on reasonable request.

## Abbreviations

ACD: Anterior chamber depth
VAE: Very asymmetric ectasia
VAE: N—very asymmetric ectasia—normal
VAE: E—very asymmetric ectasia—ectasia
MPP: Mean pupil power
KVf/b: Keratoconus Vertex front/back
BCVf/b: Baiocchi Calossi Versaci front/back
SIf/b: Symmetry Index front/back
ICLs: Intraocular collamer lenses
CCT: Central corneal thickness
IOL: Intraocular lens
UCVA: Uncorrected visual acuity
BCVA: Best corrected visual acuity
IOP: Intraocular pressure
Kmax: Maximal anterior corneal surface curvature
K1: Flat meridian corneal curvature
K2: Steep meridian corneal curvature
SE: Spherical equivalent
TCT: Thinnest corneal thickness
ACA: Anterior chamber angle
CV: Corneal volume
